# Supplementary material for: MALAT1-dependent hsa_circ_0076611 regulates translation rate in triple-negative breast cancer
Source: Commun Biol. 2022 Jun 16;5:598. doi: 10.1038/s42003-022-03539-x (PMC9203778; doi:10.1038/s42003-022-03539-x)

Supplementary Figures and Tables

MALAT1-dependent hsa_circ_0076611 regulates translation rate in triple-negative breast cancer

Chiara Turco[1], Gabriella Esposito[1], Alessia Iaiza[2], Frauke Goeman[3], Anna Benedetti[2], Enzo Gallo[4], Theodora Daralioti[4], Letizia Perracchio[4], Andrea Sacconi[5], Patrizia Pasanisi[6], Paola Muti[7,8], Claudio Pulito[1], Sabrina Strano[3], Zaira Ianniello[9], Alessandro Fatica[9], Mattia Forcato[10], Francesco Fazi[2,*], Giovanni Blandino[1,*], Giulia Fontemaggi[1,*]

[1]Oncogenomic and Epigenetic Unit, IRCCS Regina Elena National Cancer Institute, Rome, 00144, Italy
[2]Department of Anatomical, Histological, Forensic & Orthopedic Sciences, Section of Histology & Medical Embryology, Sapienza University of Rome, laboratory affiliated to Istituto Pasteur Italia-Fondazione Cenci Bolognetti, Rome, Italy;
[3]UOSD SAFU, Department of Research, Advanced Diagnostics, and Technological Innovation, Translational Research Area, IRCCS Regina Elena National Cancer Institute, Rome, Italy.
[4]Department of Pathology, IRCCS Regina Elena National Cancer Institute, Rome, 00144, Italy
[5]UOSD Clinical Trial Center, Biostatistics and Bioinformatics, IRCCS Regina Elena National Cancer Institute, Rome, 00144, Italy
[6]Unit of Epidemiology and Prevention, Fondazione IRCCS Istituto Nazionale dei Tumori, Milan, Italy
[7]Department of Health Research Methods, Evidence, and Impact, Faculty of Health Sciences, McMaster University, Hamilton, Ontario, Canada.
[8]Department of Biomedical, Surgical and Dental Sciences, "Università degli Studi di Milano", Milan, Italy.
[9]Department of Biology and Biotechnology ‘Charles Darwin’, Sapienza University of Rome, Rome, Italy
[10]Department of Life Sciences, University of Modena and Reggio Emilia, Modena, Italy

Supplementary Figure 1

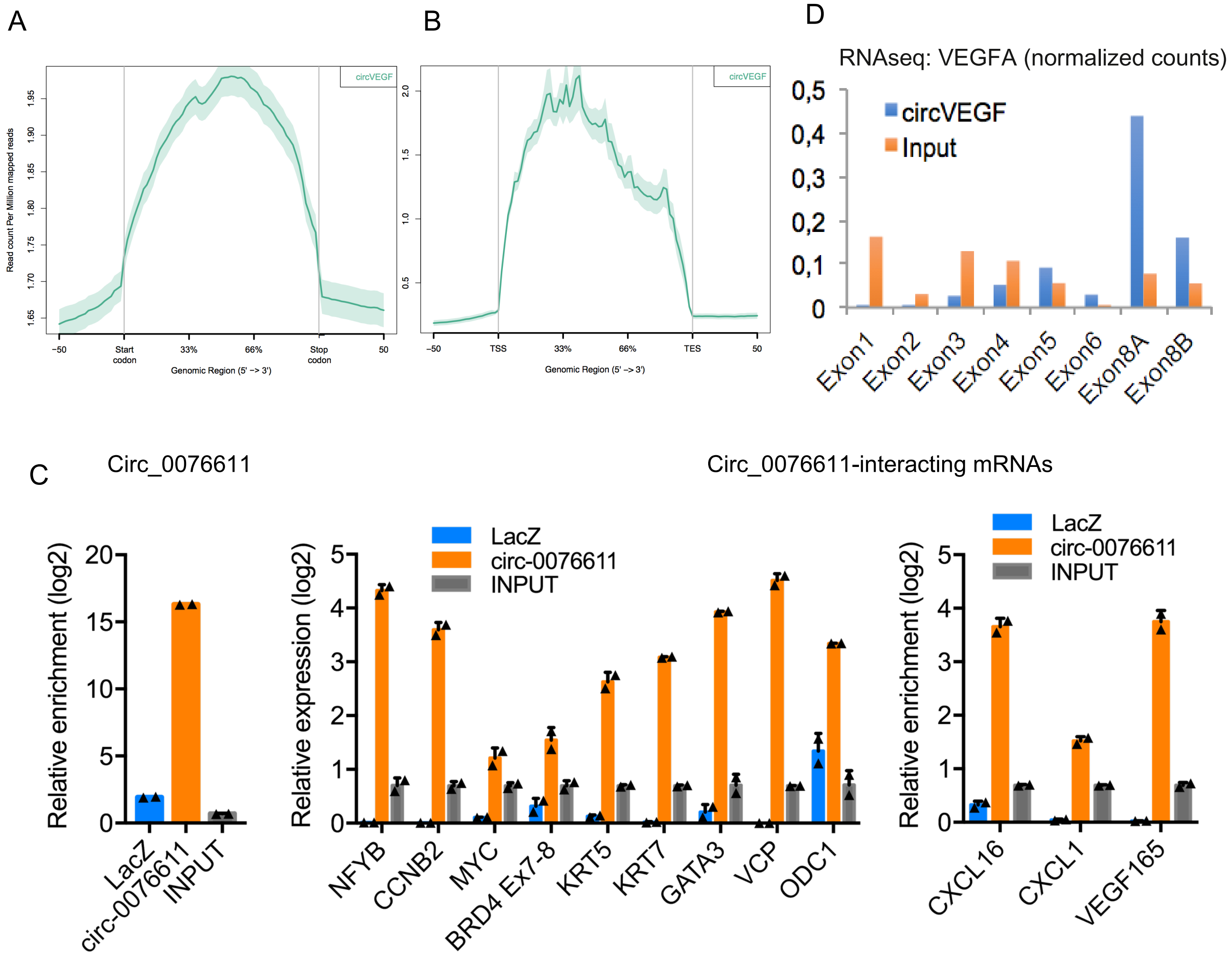

Supplementary Figure 1.

A-B. Distribution of the mean signal profile of ChIRP-RNAseq reads relatively to the coding sequence (from start codon to stop codon)(A) or to the gene body (from transcription start to transcription end)(B) of target mRNAs.
C.ChIRP experiments performed in HCC1954 cells to recover circ-0076611 (left) and its target mRNAs (middle, right)(n=2).
D. Graph reporting the ChIRP-RNAseq result of circ-0076611 for individual VEGFA exons after removal of signal from VEGFA exon 7. Exon counts have been normalized relatively to VEGFA expression.

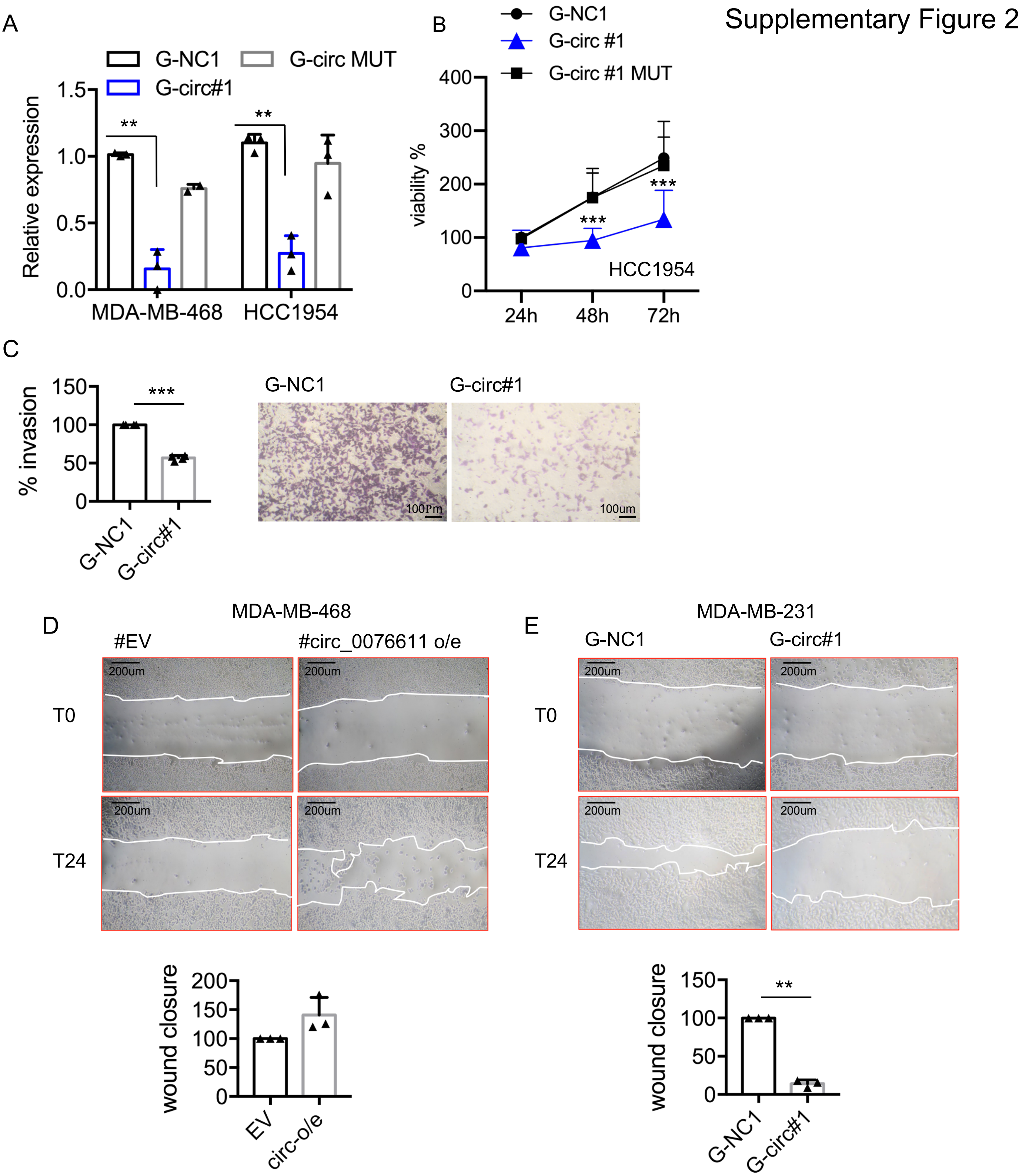

Supplementary Figure 2. A. RT-qPCR of circ-0076611 in MDA-MB-468 and HCC1954 cells, transfected with circ_0076611 GapmeR oligonucleotide (G-circ#1) or control GapmeR G-circ#1 MUT, carrying a 5-nt substitution, or control GapmeR G-NC1, and used for the viability assays shown in Figure 3H and S2B, respectively (n=3). B. Viability evaluated using ATPlite assay.
C. Invasion assay perfomed by transwell assay using matrigel-coated inserts with MDA-MB-231 cells transfected with GapmeR antisense oligonucleotide directed to circ_0076611 (G-circ#1) or control GapmeR (G-NC1).
D. Wound healing migration assay perfomed on control (EV) and circ_0076611-overexpressing (o/e) MDA-MB-468, evaluated at 24h (T24). E. Wound healing migration assay perfomed in MDA-MB-231 cells transfected with GapmeR antisense oligonucleotide directed to circ_0076611 (G-circ#1) or control GapmeR (G-NC1), evaluated at 24h (T24).
The results of at least three biological replicates are shown. $^{**}P \leq 0.005$; $^{***}P \leq 0.0005$ (paired, two-tailed Student's t-test).

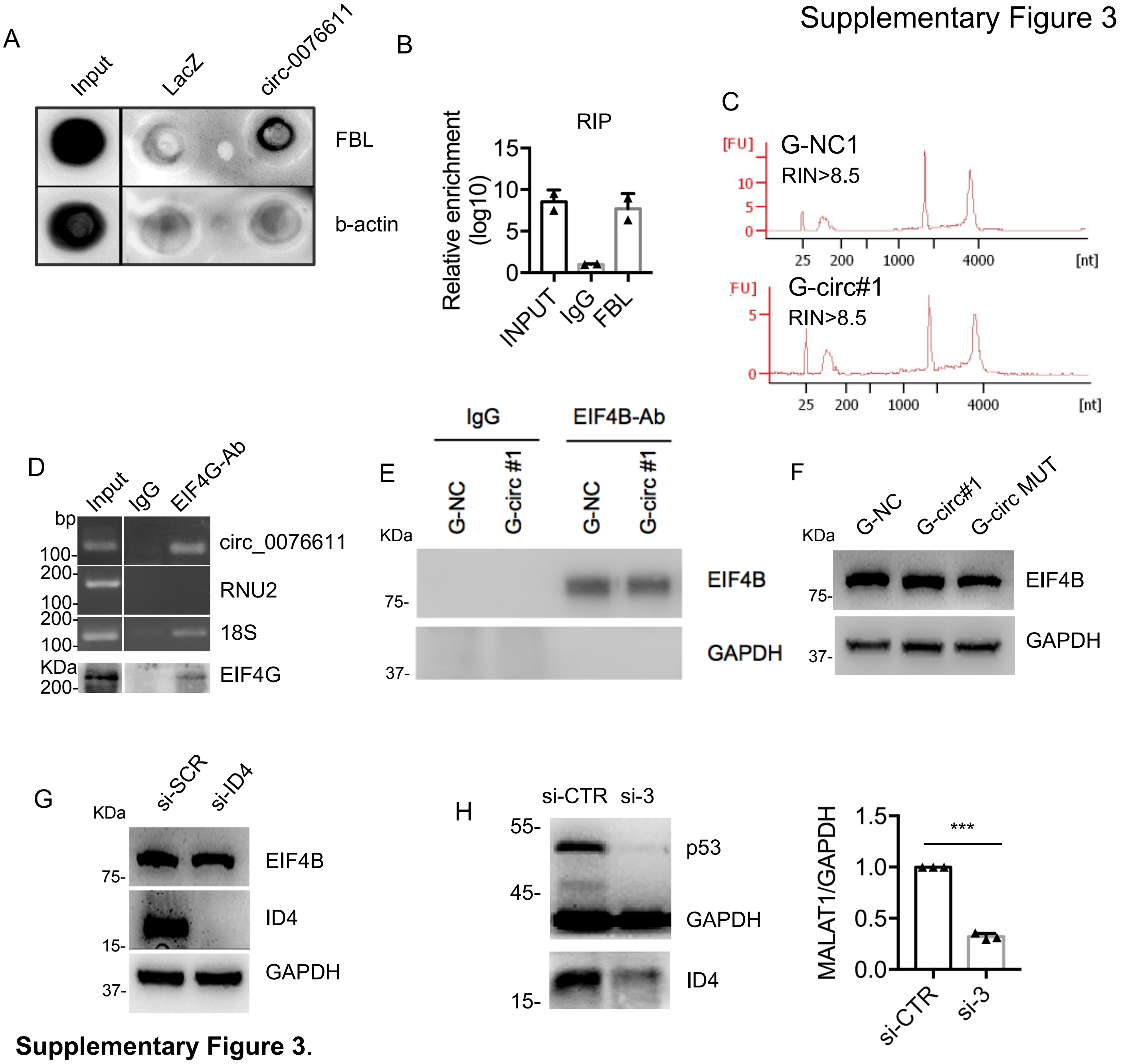

Supplementary Figure 3.
A. Dot blot analysis of fibrillarin (FBL) and beta-actin proteins on eluates from ChIRP experiments performed in MDA-MB-468, using a circ-0076611 antisense biotinylated oligonucleotide or a control LacZ oligonucleotide. Input sample has been included as positive control.
B. RT-qPCR of circ-0076611 in RIP experiments performed in MDA-MB-468 lysates by using an antibody directed to fibrillarin (FBL) or IgG as negative control (N=2). Data are normalized over RNU2 expression and presented as folds of enrichment over IgG sample.
C. Representative images of bioanalyzer electropherograms of total RNA from control (G-NC1) and circ-0076611-depleted (G-circ#1) MDA-MB-468 cells obtained using RNA 600 nano kit (Agilent Technologies).
D. Representative RIP assay in MDA-MB-468 cells crosslinked with formaldehyde using an antibody directed to eIF4G.
E. A fraction (25%) of eluates recovered during RIP experiments presented in Figure 5J has been used for protein analysis by WB to control recovery of EIF4B in control (G-NC) and circ-0076611-depleted (G-circ#1) MDA-MB-468 cells. F-G. Western blot analysis of the indicated proteins in MDA-MB-468 cells depleted of circ_0076611 (left) or of ID4 (right) expression by siRNA transfection for 72h. H. Expression of ID4 and mutant p53 proteins (left) as well as of lncRNA MALAT1 (right) has been evaluated by western blot (left) and RT-qPCR (right) in MDA-MB-468 cells transfected with siRNAs to ID4 and mutant p53, and with ASO to MALAT1 (si-3) or control oligonucleotides (si-CTR) for 48h.

A

	position	length	sequence
Stem_1_5prime	45 ~ 103	59	ctggtccttccctggctctcatcctcctggcccgtgtctctctctcactctctcactcc
Stem_1_3prime	1285 ~ 1347	63	ggagagagagagagaaagagagtgagcgagcgagcgagcgggagagcgcctgagaggggccag
Stem_2_5prime	44 ~ 103	60	cctggtccttccctggctctcatcctcctggcccgtgtctctctctcactctctcactcc
Stem_2_3prime	1285 ~ 1350	66	ggagagagagagagaaagagagtgagcgagcgagcgagcgggagagcgcctgagaggggccagctg
Stem_3_5prime	828 ~ 880	53	gagacacagcattgccccttatggcagcctctccctgcactctctgcccgtct
Stem_3_3prime	1309 ~ 1357	49	agcgagcgagcgagcgggagagcgcctgagaggggccagctgcttgctc
Stem_4_5prime	156 ~ 184	29	ggggtgaatggggtgccgacttggcctg
Stem_4_3prime	2411 ~ 2438	28	cagggccaaggcacccccagctcacccc
Stem_5_5prime	652 ~ 687	36	agcctcccatcacaccctactttagcccaccttggt
Stem_5_3prime	2576 ~ 2611	36	accaagagggcgtatattagtgctggggtgggggct
Stem_6_5prime	796 ~ 818	23	ccctgtggcctgagactcaccct
Stem_6_3prime	2635 ~ 2657	23	aggggagtctcagttccccaggg
Stem_7_5prime	610 ~ 637	28	tggccaccagcggcctggcctggggaca
Stem_7_3prime	3159 ~ 3182	24	tgtccccagggcaggcccttgcca
Stem_8_5prime	1300 ~ 1338	39	aagagagtgagcgagcgagcgagcgggagagcgcctgag
Stem_8_3prime	3212 ~ 3247	36	ctcaggcgttctcccagaagatctgcccactctctt
Stem_9_5prime	1860 ~ 1913	54	ccccgctcagactgaggctccctcaggccagggctatgtctccctcctcagact
Stem_9_3prime	3436 ~ 3489	54	agctcaggagggtagactgggagcccctgagtggagctgctgctcaggccgggg
Stem_10_5prime	593 ~ 628	36	ccctgccctgggccctctggccaccagcggcctggc
Stem_10_3prime	3477 ~ 3510	34	gctcaggccggggctccctgagggcagggctggg

B

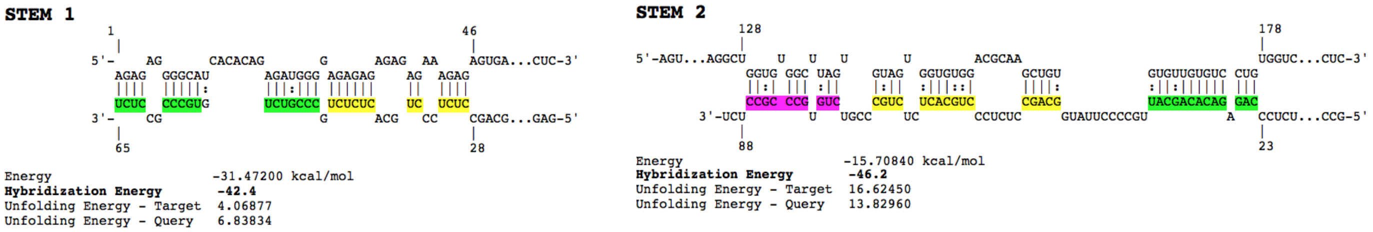

C

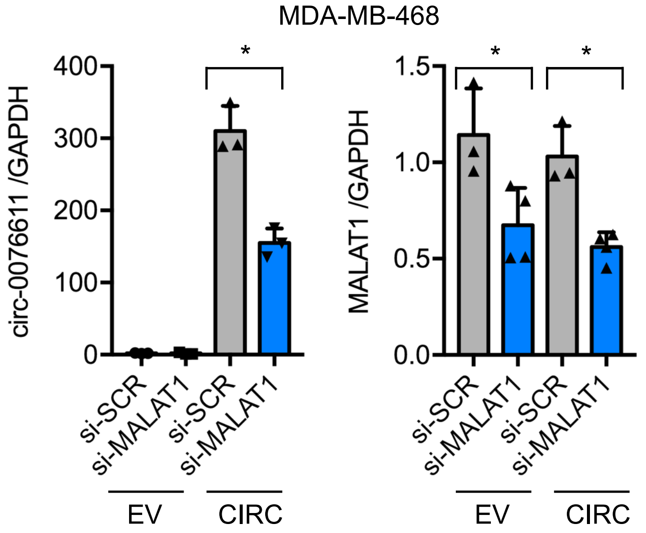

D

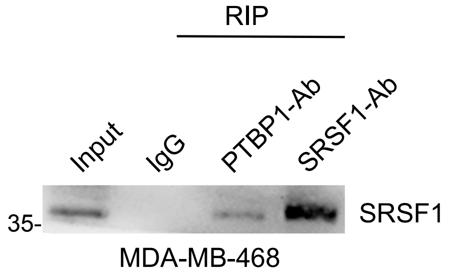

Supplementary Figure 4.
A. Inverted-repeated sequences, potentially involved in the formation of stem-loop structures, present in the introns flanking VEGFA exon 7 (intron 6 and 7) were initially identified using RegRNA 2.0 tool (http://regrna2.mbc.nctu.edu.tw/).
B. The prediction of hybridization for the two couples of inverted-repeated sequences closest to exon 7, identified by RegRNA analysis, and included in the fragment of VEGFA gene cloned in the circ-0076611 expression vector, has been carried out using the IntaRNA bioinformatic tool (http://rna.informatik.uni-freiburg.de/IntaRNA).
C. Expression of circ_0076611 (left) and MALAT1 (right), evaluated by RT-qPCR in MDA-MB-468 cells transiently transfected with a pCDNA3.1 vector (EV) or with the circ_0076611 expression vector (CIRC), in presence (si-SCR) or absence (si-MALAT1) of MALAT1.
D. Western blot analysis of SRSF1 on proteins recovered using SRSF1-Ab and PTBP1-Ab in RIP experiment in MDA-MB-468.

```
           gcctgagactcacccttgtctcccagagacacagcattgc
cccttatggcagcctctccctgcactctctgcccgtctgtgcccgcctct
tcctgcggcaggtgtcctagccagtgctgcctctttccgccgctctctct   Intron 6
gtcttttgctgtagcgctcggatccttccagggcctgggggctgaccggc
tgggtgggggtgcagctgcggacatgttaggggtgttgcatggtgattt
tttttctctctctctgctgatgctctagcttagatgtctttcctttgcc
tttttgcagTCCCTGTGGGCCTTGCTCAGAGCGGAGAAAGCATTTGTTTG
TACAAGATCCGCAGACGTGTAAATGTTCCTGCAAAAACACAGACTCGCGT   Exon 7
TGCAAGGCGAGGCAGCTTGAGTTAAACGAACGTACTTGCAGgttggttcc
cagagggcaagcaagtcagagaggggcatcacacagagatggggagagag
agagagaaagagagtgagcgagcgagcgagcgggagagcgcctgagaggg
gccagctgcttgctcagtttctagctgcctgcctggtgactgctgccttc   Intron 7
tctgcttttaaggcccctgtggtgggctgcaggcactggtccagcctggc
ggggcctgttccgaggttgccctggttgcctgagtggtaggctggtgtgg
cttagtgtagtggtgtggacgcaagctgtgtgttgtgtcctgtggtcctt
ctgctc

SRSF1
PTBP1
```

Supplementary Figure 5. VEGFA genomic region cloned in pCDNA3.1 plasmid to generate a circ-0076611 expression vector. Predicted binding sites for SRSF1 and PTBP1 splicing regulators are indicated.

Supplementary Table 1. List of siRNA and GapmeR sequences used in the study.

siRNA	Sequence
circ_0076611 (IdT)	For 5′-AACGUACUUGCAGUCCCUGUGUU-3′
	Rev 5′-AACACAGGGACUGCAAGUACGUU-3′
MALAT1#1 (IdT hs.Ri.MALAT1.13.1)	For 5′-GCUCCUUGGUGAAUUGAUAAGUAAA-3′
	Rev 5′-UUUACUUAUCAAUUCACCAAGGAGCUG-3′
MALAT1#2	5'-GATCCATAATCGGTTTCAA-3'
ASO-MALAT1	mG*mG*mG*mA*mG*T*T*A*C*T*T*G*C*C*A*mA*mC*mU*mU*mG (from Tripathi, Mol Cell 2010)
ASO-SCR	mC*mU*mA*mU*mA*A*C*G*G*C*G*C*T*C*G*mA*mU*mG*mA*mU
SRSF1#1	5'-GAAAGAAGATATGACCTAT-3'
SRSF1#2	5'-TGAAGCAGGTGATGTATGT-3'
ID4-siRNA	For 5′-GATCCTGCAGCACGTTATC-3′
	Rev 5′-TTACAGAGCTCTTGATATC-3′
p53_siRNA	5'-GACUCCAGUGGUAAUCUAC-3' (Brummelkamp et al 2002; doi: 10.1126/science.1068999)
si-PTBP1	Trifecta RNAi kit (IdT) hs.Ri.PTBP1.13
si-SCR for 27-mer	Negative Control DsiRNA (IdT)
si-SCR for 21-mer	5'-CTATAACGGCGCTCGATAT-3'
**GapmeR**	**Sequence**
G-circ	5′-CAGGGACTGCAAGTAC-3′
G-circMUT	5′-CAGGTCAGCCAAGTAC-3′
G-NC	5'-AACACGTCTATACGC-3'

Uncropped gels relative to Figure 1

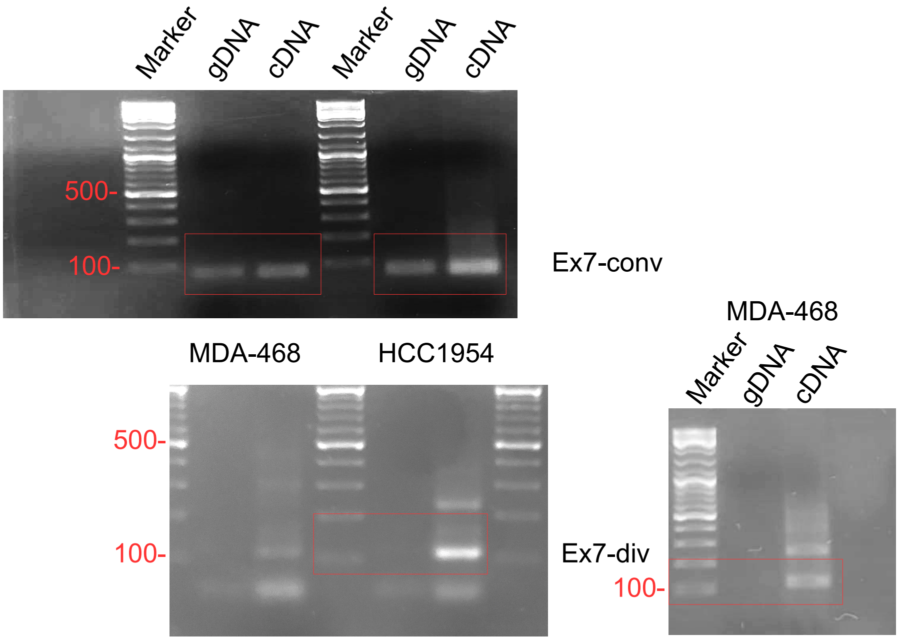

Uncropped blots relative to Figure 2G

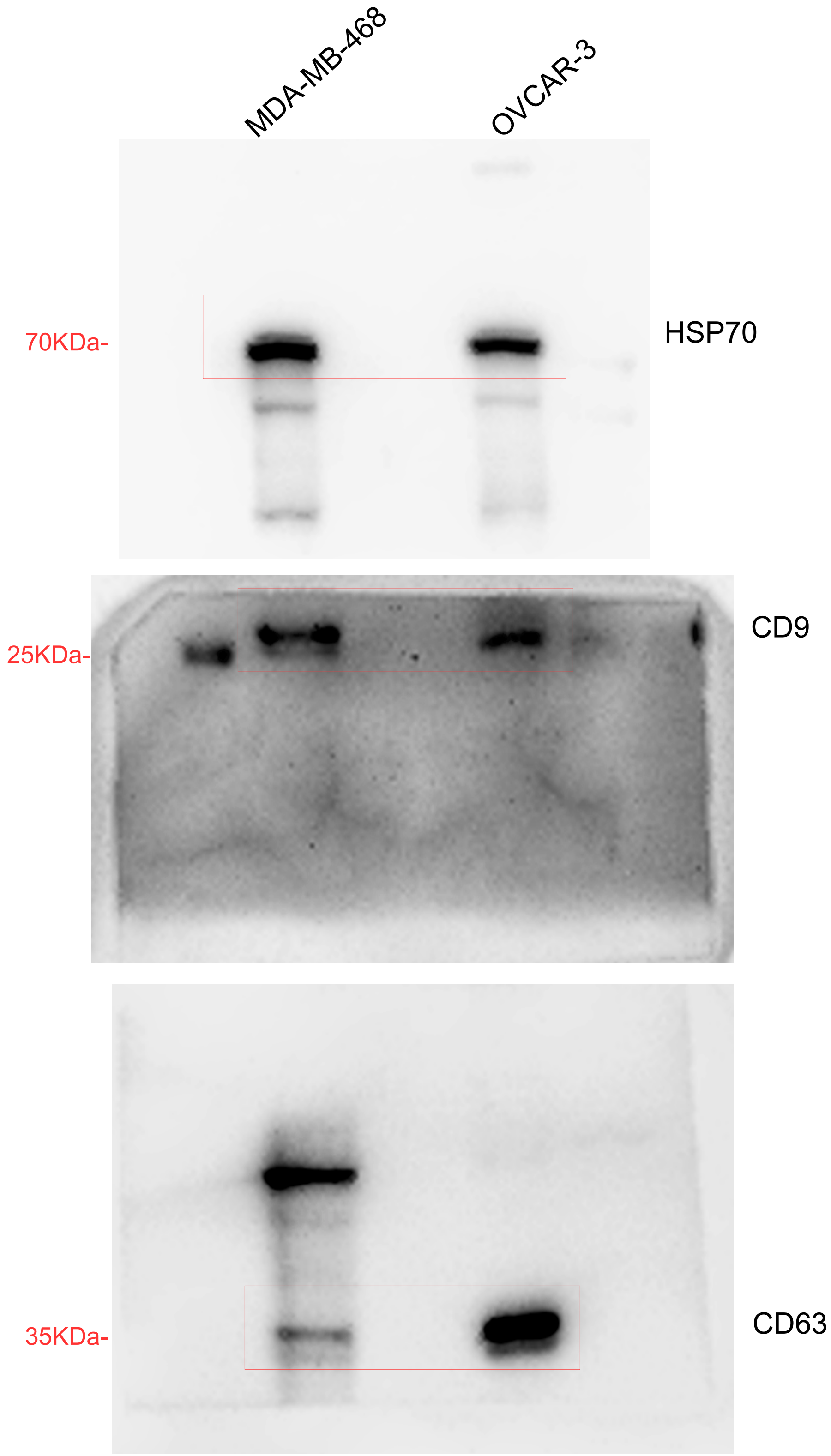

Uncropped blots relative to Figure 3J

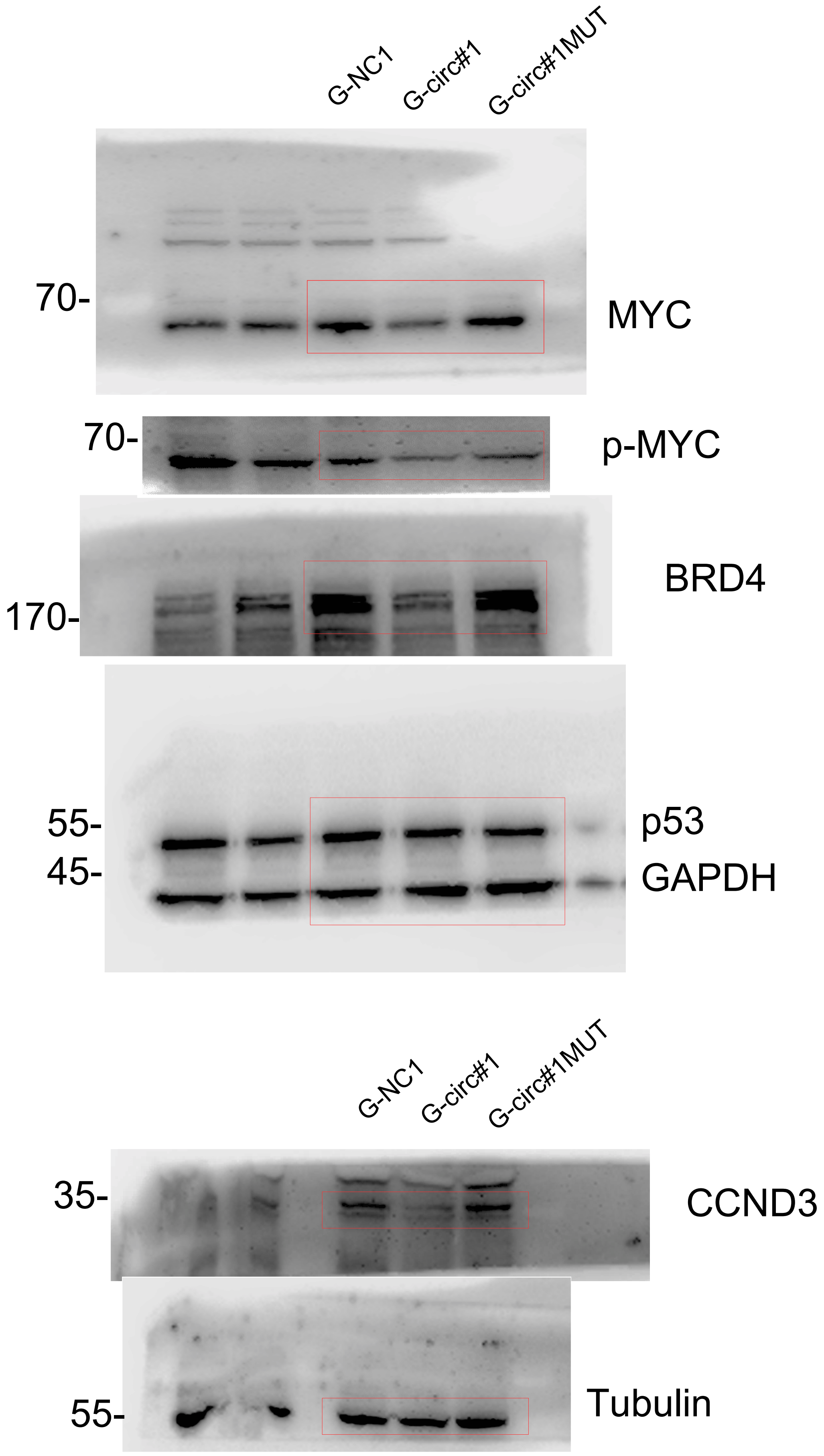

Uncropped blots relative to Figure 3K

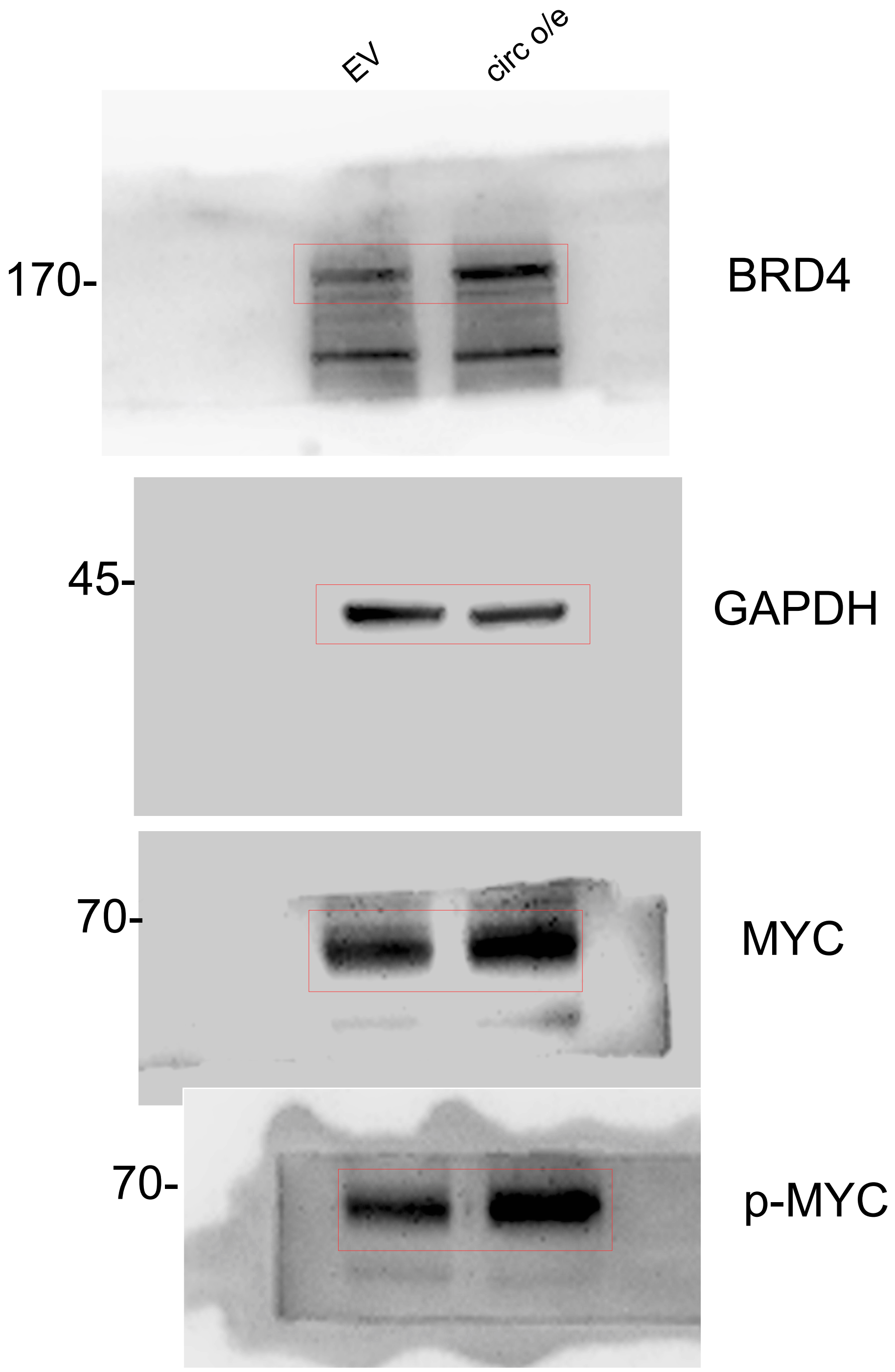

Uncropped blots relative to Figure 4E-F

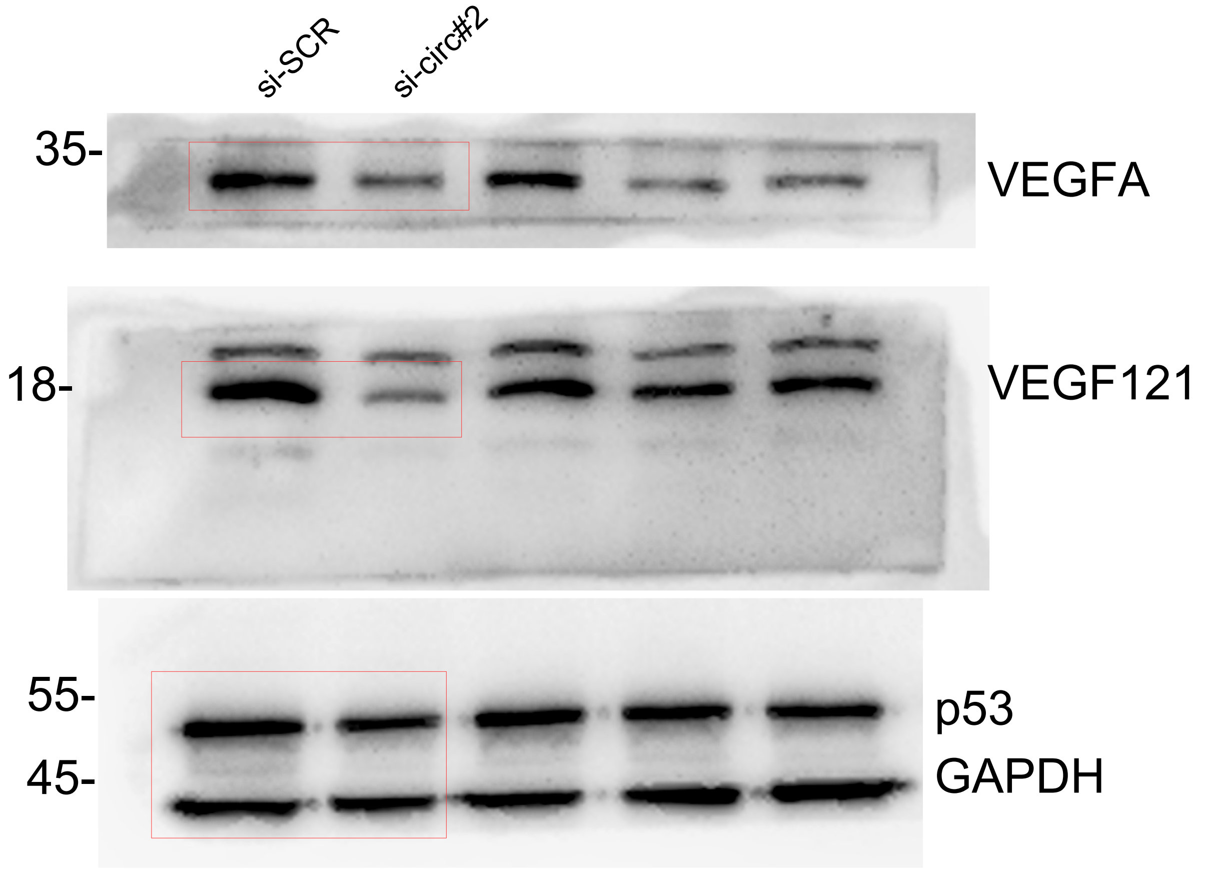

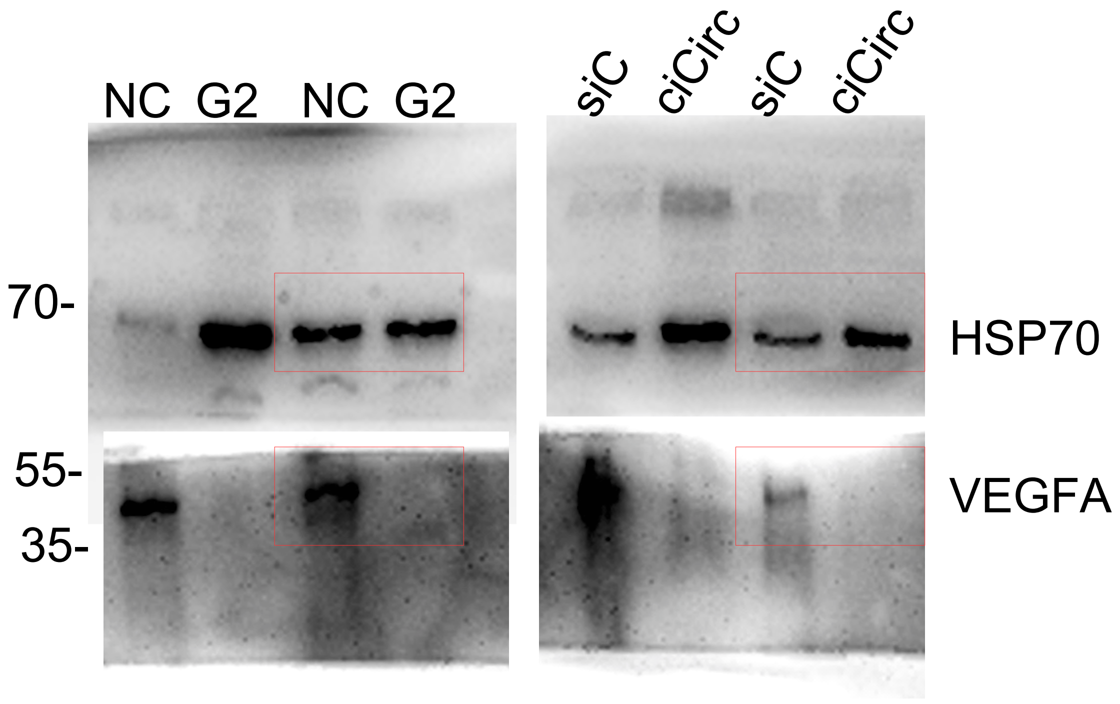

Uncropped blots relative to Figure 5

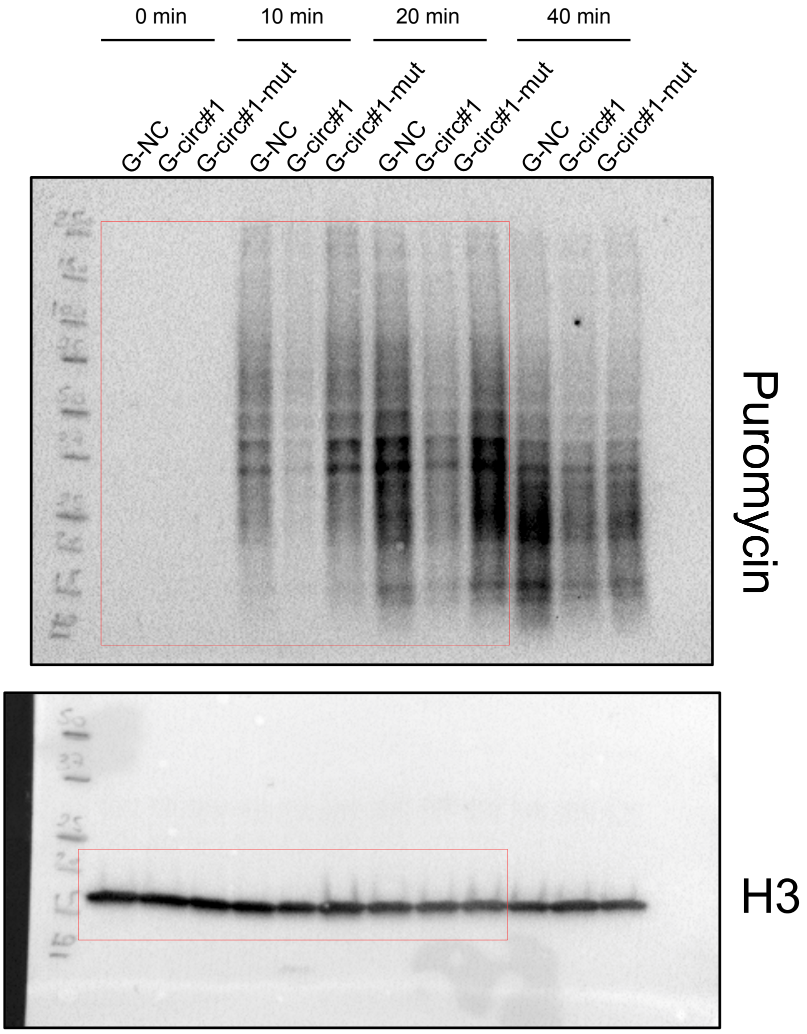

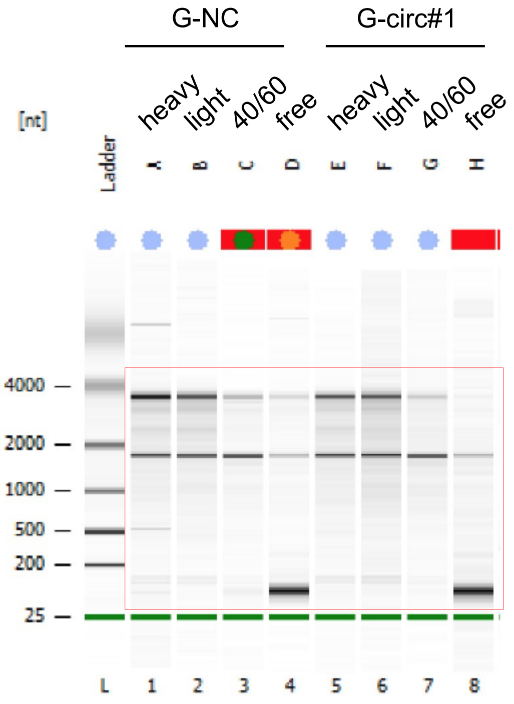

Uncropped gels relative to Figure 6

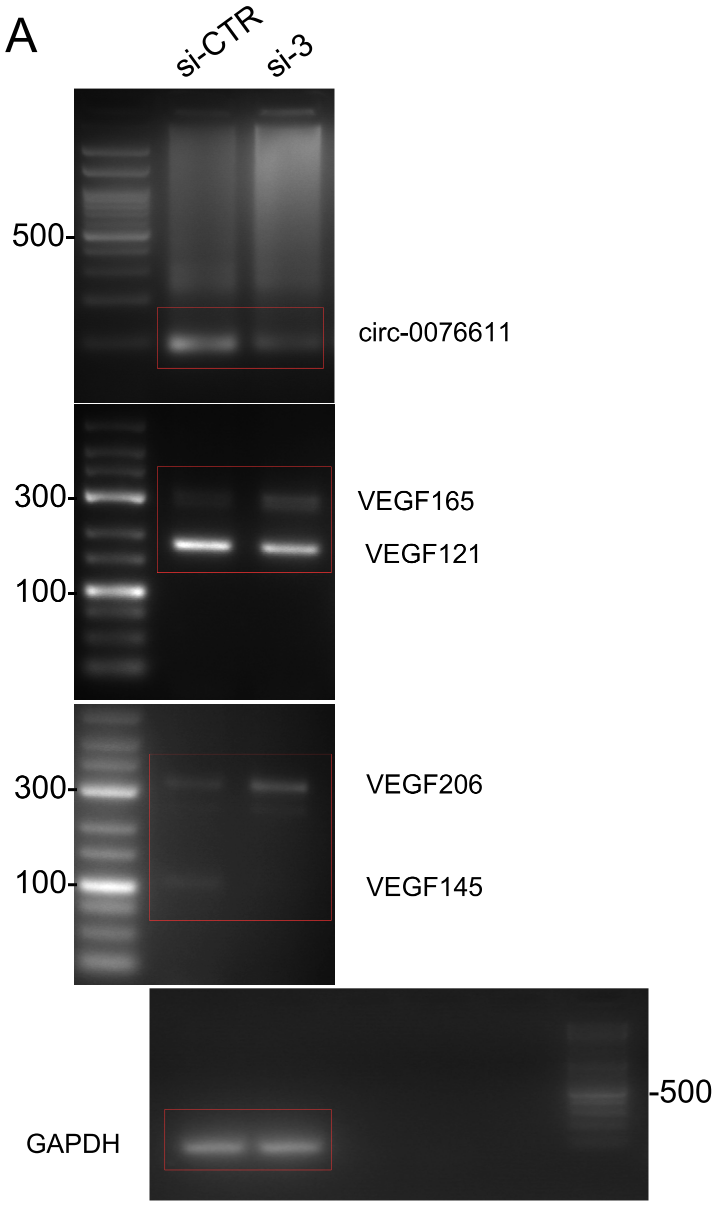

Uncropped blots relative to Figure 6

F

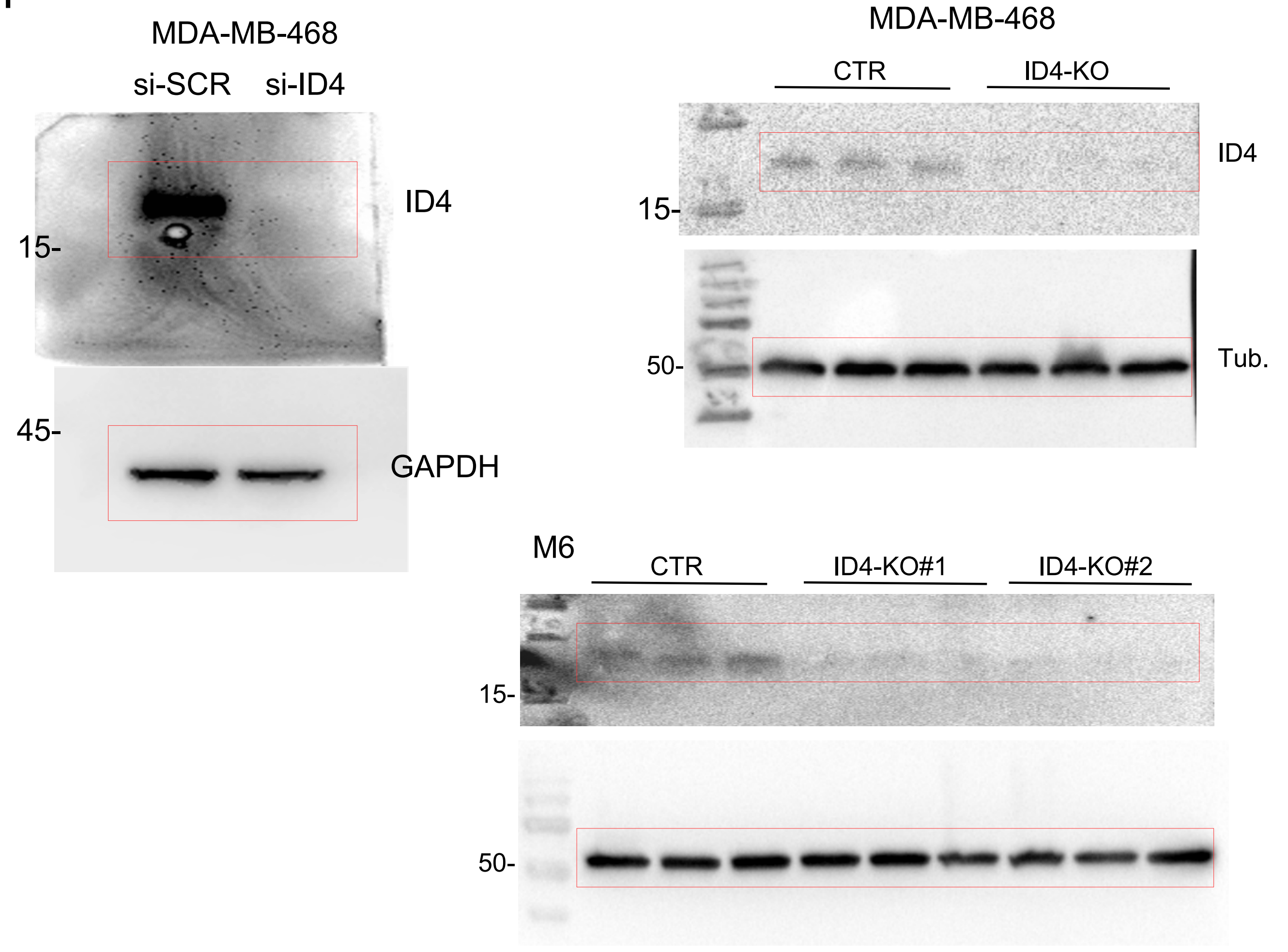

G

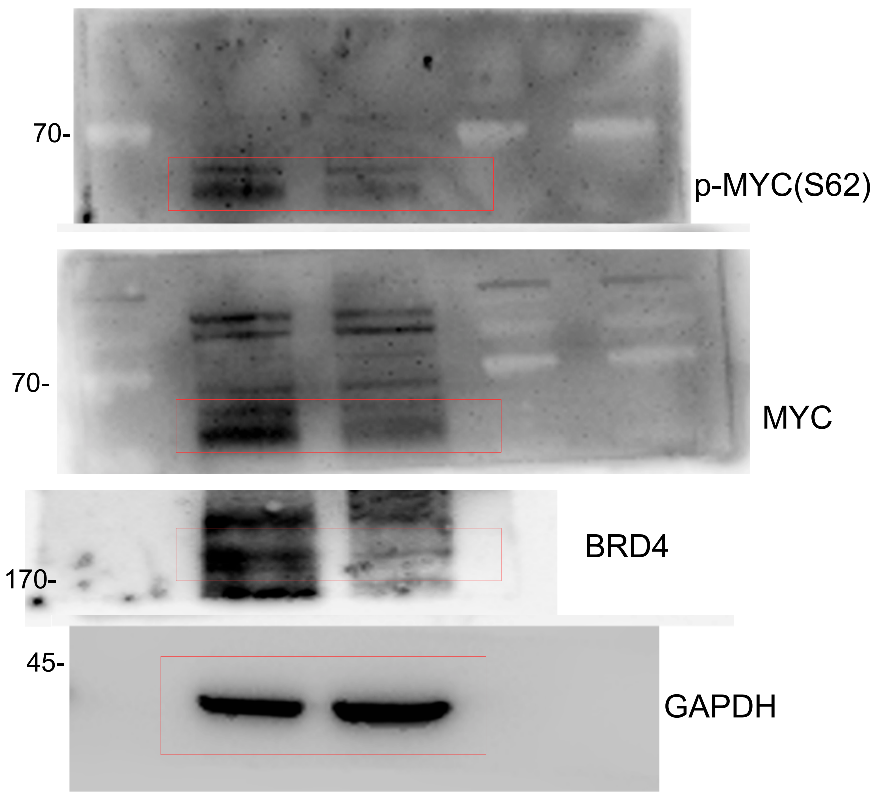

Uncropped blots relative to Figure 7C

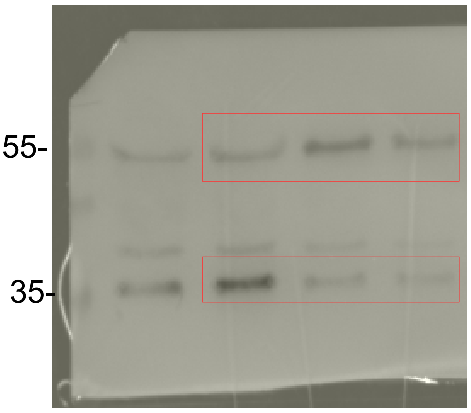

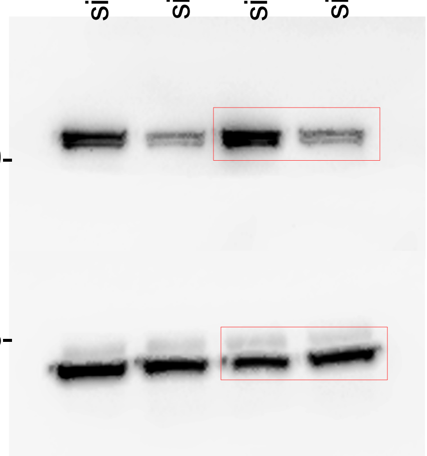

Uncropped blots relative to Figure 7E

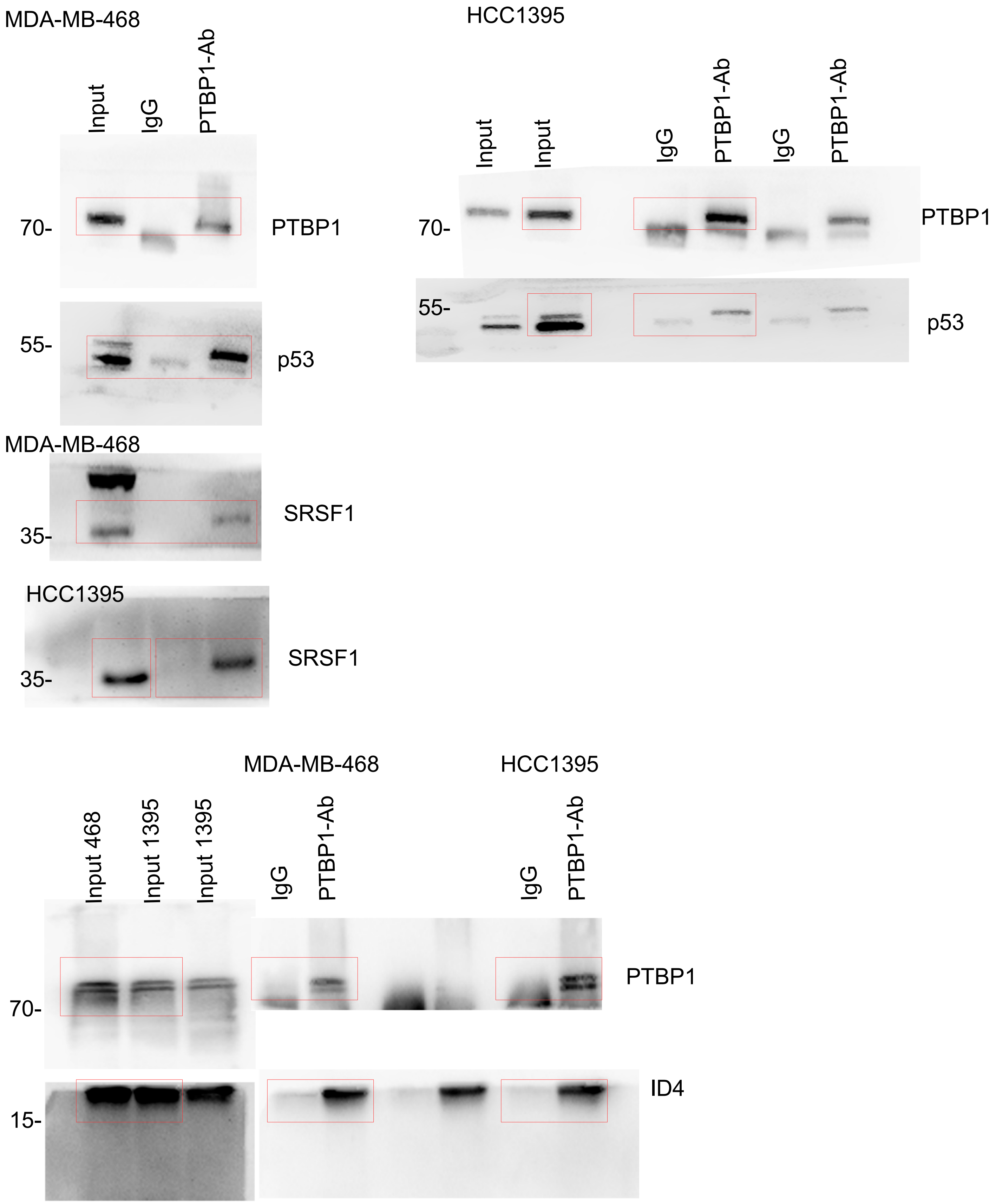

Supplement: Supplementary file 2 — Supplementary Information [file 42003_2022_3539_MOESM2_ESM.pdf]
