# Supplementary material for: MALAT1-dependent hsa_circ_0076611 regulates translation rate in triple-negative breast cancer
Source: Commun Biol. 2022 Jun 16;5:598. doi: 10.1038/s42003-022-03539-x (PMC9203778; doi:10.1038/s42003-022-03539-x)
Supplement: Supplementary file 3 — Description of Additional Supplementary Files [file 42003_2022_3539_MOESM3_ESM.pdf]

Description of Additional Supplementary Files

File name: Supplementary Data 1
Description: List of oligonucleotides used in PCR/qPCR and for the gene editing of human and mouse ID4 by CRISPR/Cas9.
