# Supplementary material for: MALAT1-dependent hsa_circ_0076611 regulates translation rate in triple-negative breast cancer
Source: Commun Biol. 2022 Jun 16;5:598. doi: 10.1038/s42003-022-03539-x (PMC9203778; doi:10.1038/s42003-022-03539-x)
Supplement: Supplementary file 4 — Supplementary Data 1 [file 42003_2022_3539_MOESM4_ESM.pdf]

Supplementary Data File 1. List of oligonucleotides used in PCR/qPCR and for the gene editing of human and mouse ID4 by CRISPR/Cas9

**RT-qPCR**	**Sequence**
**VEGF121**	
Ex3_F	5′-CCCACTGAGGAGTCCAACAT-3′
Ex5-8_R	5′-CCTCGGCTTGTCACATTTTTCTTGTC-3′
Ex4_probe_FAM	5′-AAGGCCAGCACATAGGAGAGAT-3′
**VEGF165**	
Ex7_F	5′-CGCAGACGTGTAAATGTTCCT-3′
Ex8_R	5′-GCCTCGGCTTGTCACATC-3′
Ex7_probe_FAM	5′-CAAGGCGAGGCAGCTTGAGTTAAA-3′
**Circular VEGFA**	
Ex7_div_F	5'-CGCAGACGTGTAAATGTTCCT-3'
Ex7_div_R	5'-AGGAACATTTACACGTCTGCG-3'
Ex7_div_F2	5'-AAACACAGACTCGCGTTGC-3'
Ex7_div_R2	5'-CAAATGCTTTCTCCGCTCTG-3'
circ_0076611 Taqman probe	/5'6-FAM/AA CGT ACT T/ZEN/G CAG TCC CTG T/3'IABkFQ/
**VEGFA**	
Ex7_conv_F	5'-GAGCGGAGAAAGCATTTGTT-3'
Ex7_conv_R	5'-GCGAGTCTGTGTTTTTGCAG-3'
5′UTR_F	5′-GTCGAGGAAGAGAGAGACGG-3′
5′UTR_R	5′-CCCAAAAGCAGGTCACTCAC-3′
**ID4**	
For	5′-GTGCGATATGAACGACTGCT-3′
Rev	5′-CAGGATCTCCACTTTGCTGA-3′
**MALAT1**	
For	5′-GGGAAGGCGAAGAAAAGAAT-3′
Rev	5′-TGCCCTTAGCTTTTTGTTTCC-3′
**PTBP1**	
For	5'-GCTGCACCTCTCCAACATCC-3'
Rev	5'-GTCGTGGTTGTGCAGGTCAA-3'
**c-MYC**	
Ex2_F	5′-AGCTGCTTAGACGCTGGATT-3′
Ex2_R	5′-AAGTTCTCCTCCTCGTCGC-3′
5′UTR_F	5′-CATCCACGAAACTTTGCCCA-3′
5′UTR_R	5′-GCTCGGGTGTTGTAAGTTCC-3′
**CXCL16**	
Ex3_F	5′-CTTTCCTGGAGCGTGTGTG-3′
Ex4_R	5′-GTAAATGCTTCTGGTGGGCC-3′
**CXCL1**	

Ex1_F	5′-CTCTTCCGCTCCTCTCACAG-3′
Ex2_R	5′-GGGGACTTCACGTTCACACT-3′
**18S**	
For	5′-AGCATTTGCCAAGAATGTTTTC-3′
Rev	5′-CGTCTTCGAACCTCCGACTT-3′
**28S**	
Fw	5′-GTTCACCCACTAATAGGGAACG-3′
Rev	5′-GGATTCTGACTTAGAGGCGTT-3′
**5.8S**	
For	5′-GACTCTTAGCGGTGGATCACTC-3′
Rev	5′-GACGCTCAGACAGGCGTAG-3′
**BRD4**	
Ex7_F	5′-ACGTCCGATTGATGTTCTCC-3′
Ex8_R1	5′-AAAGCGCATTTCGAACACAT-3′
Ex8_F	5′-CGATAGCTCCTCGGACAGTG-3′
Ex8_R2	5′-CGCTCCTCCTCAGAGTCATC-3′
**CCNB2**	
For	5′-TGCAAAATCGAGGACATTGA-3′
Rev	5′-TGTGGGTTTATGGACTGCAA-3′
5′UTR_F	5′-TGTCCTCCCTTTTCAGTCCG-3′
5′UTR_R	5′-GACGGGGAAGGCAAGAGT-3′
**NFYB**	
For	5′-GTTTGCGGTCCCTGTACTTG-3′
Rev	5′-GAACCGTGTTGTCAGTGGTG-3′
**GAPDH**	
For	5′-GAGTCAACGGATTTGGTCGT-3′
Rev	5′-GACAAGCTTCCCGTTCTCAG-3′
**RPL19**	
For	5′-CGGAAGGGCAGGCACAT-3′
Rev	5′-GGCGCAAAATCCTCATTCTC-3′
**CRISPR-Cas9 oligonucleotides**	**Sequence**
Human	
Hs.Cas9.ID4.1.AA	5′-GCACGTTATCGACTACATCC-3′; PAM: TGG
Hs.Cas9.ID4.1.AD	5′-TGTAGTCGATAACGTGCTGC-3′; PAM: AGG
Mouse	
Hs.Cas9.ID4.1.AA	5′-GCACGTTATCGACTACATCC-3′; PAM: TGG
Mm.Cas9.ID4.1.AB	5′-GAGCACGGCCACAGCCTGGG-3′; PAM: TGG

**Pull-down**	**Sequence**
Bio_circ_0076611	CAGGGACTGCAAGTAC/3BioTEG/
Bio_LacZ_1	GTCATATGCATAAAGCGTTG/3BioTEG/
Bio_LacZ_2	TTAACGCCGCAGTGGTAGAA/3BioTEG/
