# Supplementary material for: MALAT1-dependent hsa_circ_0076611 regulates translation rate in triple-negative breast cancer
Source: Commun Biol. 2022 Jun 16;5:598. doi: 10.1038/s42003-022-03539-x (PMC9203778; doi:10.1038/s42003-022-03539-x)
Supplement: Supplementary file 5 — Reporting Summary [file 42003_2022_3539_MOESM5_ESM.pdf]

nature research

Corresponding author(s): Giulia Fontemaggi
Giovanni Blandino
Francesco Fazi

Last updated by author(s): 13/04/2022

Reporting Summary

Nature Research wishes to improve the reproducibility of the work that we publish. This form provides structure for consistency and transparency in reporting. For further information on Nature Research policies, see our Editorial Policies and the Editorial Policy Checklist.

Statistics

For all statistical analyses, confirm that the following items are present in the figure legend, table legend, main text, or Methods section.

n/a	Confirmed	
☐	☒	The exact sample size (n) for each experimental group/condition, given as a discrete number and unit of measurement
☐	☒	A statement on whether measurements were taken from distinct samples or whether the same sample was measured repeatedly
☐	☒	The statistical test(s) used AND whether they are one- or two-sided<br>*Only common tests should be described solely by name; describe more complex techniques in the Methods section.*
☒	☐	A description of all covariates tested
☒	☐	A description of any assumptions or corrections, such as tests of normality and adjustment for multiple comparisons
☒	☐	A full description of the statistical parameters including central tendency (e.g. means) or other basic estimates (e.g. regression coefficient) AND variation (e.g. standard deviation) or associated estimates of uncertainty (e.g. confidence intervals)
☒	☐	For null hypothesis testing, the test statistic (e.g. F, t, r) with confidence intervals, effect sizes, degrees of freedom and P value noted<br>*Give P values as exact values whenever suitable.*
☒	☐	For Bayesian analysis, information on the choice of priors and Markov chain Monte Carlo settings
☒	☐	For hierarchical and complex designs, identification of the appropriate level for tests and full reporting of outcomes
☒	☐	Estimates of effect sizes (e.g. Cohen's d, Pearson's r), indicating how they were calculated

Our web collection on statistics for biologists contains articles on many of the points above.

Software and code

Policy information about availability of computer code

Data collection	No software was used
Data analysis	No software was used

For manuscripts utilizing custom algorithms or software that are central to the research but not yet described in published literature, software must be made available to editors and reviewers. We strongly encourage code deposition in a community repository (e.g. GitHub). See the Nature Research guidelines for submitting code & software for further information.

Data

Policy information about availability of data

All manuscripts must include a data availability statement. This statement should provide the following information, where applicable:

- Accession codes, unique identifiers, or web links for publicly available datasets
- A list of figures that have associated raw data
- A description of any restrictions on data availability

DATA AVAILABILITY
ChIRP-RNAseq data are available in GEO database as GSE183902
Uncropped gels and blots are included as Supplementary Material
Source data for graphs are available in "Figshare"

Field-specific reporting

Please select the one below that is the best fit for your research. If you are not sure, read the appropriate sections before making your selection.

☒ Life sciences ☐ Behavioural & social sciences ☐ Ecological, evolutionary & environmental sciences

For a reference copy of the document with all sections, see nature.com/documents/nr-reporting-summary-flat.pdf

Life sciences study design

All studies must disclose on these points even when the disclosure is negative.

Sample size	As no measurement of hsa_circ_0076611 had been previously performed in the literature, we couldn't carry out a sample size calculation to analyze the associations between hsa_circ_0076611 expression and clinical variables or ID4 protein expression. Therefore we chose to analyze a series of triple-negative breast cancer (TNBC) cases because the over expression of ID4 had been reported specifically in this subtype. The choice of TNBC allowed to reduce the variability determined by the receptor status in breast cancer.<br>A tissue microarray enclosing 116 triple-negative breast cancer (TNBC) cases allowed identifying association between hsa_circ_0076611 and tumor size. Association between hsa_circ_0076611 and ID4 protein expression was instead assessed on 67 Ck+ TNBC cases.
Data exclusions	No data were excluded from the analyses
Replication	All attempts at replication were successful
Randomization	The study does not involve randomization
Blinding	The investigators were blinded during data collection of hsa_circ_0076611 and ID4 expression on TMA

Reporting for specific materials, systems and methods

We require information from authors about some types of materials, experimental systems and methods used in many studies. Here, indicate whether each material, system or method listed is relevant to your study. If you are not sure if a list item applies to your research, read the appropriate section before selecting a response.

Materials & experimental systems

n/a	Involved in the study	
☐	☒	Antibodies
☐	☒	Eukaryotic cell lines
☒	☐	Palaeontology and archaeology
☒	☐	Animals and other organisms
☐	☒	Human research participants
☐	☒	Clinical data
☒	☐	Dual use research of concern

Methods

n/a	Involved in the study	
☒	☐	ChIP-seq
☒	☐	Flow cytometry
☒	☐	MRI-based neuroimaging

Antibodies

Antibodies used	anti-ID4 (clone: B-5) (Santa Cruz)<br>anti-CK5 (clone: XM26) (Novocastra™)<br>anti-PTBP1 (Cell Signaling: #72669),<br>anti-eIF4B (Cell Signaling: #3592),<br>anti-eIF4G (Cell Signaling: #2469)<br>anti-SRSF1 (Santa Cruz: SF2/ASF (96) sc-33652)<br>anti-Fibrillarin (Abcam: FBL ab5821)<br>anti-p53 DO-1 (Cell Signaling),<br>anti-VEGFA ab46154 (Abcam),<br>anti-VEGF121 abx129570 (Abbexa),<br>anti-BRD4 A301-985A100 (Bethyl),<br>anti-CCND3 ab28283 (Abcam),<br>anti- c-Myc SC-40 (Santa Cruz),<br>anti-c-Myc-p (Ser62) E1J4K (Cell Signaling),<br>anti-α-Tubulin DM1A (Cell Signaling),<br>anti- GAPDH sc-47724 (Santa Cruz).
Validation	anti-ID4 (clone: B-5) (Santa Cruz) used for WB and IHC, validated by the analysis of ID4-KO cells of human and mouse derivation<br>anti-CK5 (clone: XM26) (Novocastra™) routinely used in breast cancer diagnostics<br>anti-PTBP1 (Cell Signaling: #72669), for RIP/IP and WB, validated by the use of siRNAs directed to PTBP1 mRNA

anti-eIF4B (Cell Signaling: #3592), for RIP/IP and WB, validated by performing IP followed by WB
anti-SRSF1 (Santa Cruz: SF2/ASF (96) sc-33652), for RIP/IP and WB, validated in our previous study by the use of siRNAs directed to SRSF1 mRNAs (Pruszko et al., EMBO Rep 2017)
anti-Fibrillarin (Abcam: FBL ab5821), for RIP/IP and dot blot, validated by performing IP followed by WB
anti-p53 DO-1 (Cell Signaling), for WB, validated by the analysis of p53-KO cells and siRNAs directed to p53
anti-VEGFA ab46154 (Abcam), for WB, validated by the use of siRNAs directed to VEGFA mRNA
anti-VEGF121 abx129570 (Abbexa), for WB
anti-BRD4 A301-985A100 (Bethyl), for WB
anti-CCND3 ab28283 (Abcam), for WB
anti- c-Myc SC-40 (Santa Cruz), for WB, validated by the use of siRNAs directed to MYC mRNA in our previous study (Ganci et al Clin Cancer Res 2021)
anti-c-Myc-p (Ser62) E1J4K (Cell Signaling), for WB, validated by the use of siRNAs directed to MYC mRNA (Ganci et al Clin Cancer Res 2021)
anti-α-Tubulin DM1A (Cell Signaling), for WB
anti- GAPDH sc-47724 (Santa Cruz), for WB

Eukaryotic cell lines

Policy information about cell lines

Cell line source(s)	MDA-MB-468 (ATCC® HTB-132)<br>MCF10A (ATCC® CRL-10317)<br>HCC1954 (ATCC® CRL2338™)<br>HCC70 (ATCC® CRL2315™)<br>HCC1395 (ATCC® SC-CRL-2324)<br>HCC1143 (ATCC® CRL-2321)<br>SK-BR-3 (ATCC® HTB-30™)<br>MDA-MB-231 (ATCC® HTB-26)<br>Ovcar-3 (ATCC® HTB-161)
Authentication	Except for MCF10A all cell lines carry point mutation of TP53 leading to mutant p53 protein expression. These cell lines were subjected to the control of TP53 mutational status periodically by Sanger sequencing of TP53 coding exons (1-11).
Mycoplasma contamination	PCR-based mycoplasma testing
Commonly misidentified lines<br>(See ICLAC register)	N/A

Human research participants

Policy information about studies involving human research participants

Population characteristics	Analysis of the expression of hsa_circ_0076611 has been performed by in situ hybridization on a commercial tissue microarray (TMA #BR1301 from US Biomax, Inc) enclosing 116 tumor tissues from patients diagnosed with breast cancer with the following characteristics: breast carcinoma with ER, PR and Her-2 all negative; Age in the 31-86 range (Mean age 52).<br>Analysis of the expression of hsa_circ_0076611 has been performed by RT-qPCR on serum samples from postmenopausal patients of age <70 years with breast cancer and without diabetes who have basal serum testosterone levels = or >0.28 ng/mL (median value) recruited for the randomized study reported by Campagnoli et al., 2012 (PMID: 22607767).<br>Analysis of the expression of hsa_circ_0076611 has been performed by RT-qPCR on a group of 12 ER-negative breast tumor and peritumor samples (fresh frozen) randomly selected.
Recruitment	No specific recruitment criteria are indicated for the commercially distributed tissue microarray (TMA #BR1301 from US Biomax, Inc) enclosing 116 tumor tissues from patients diagnosed with triple-negative breast cancer.<br>Samples of fresh frozen tumor and peritumor tissues (ER-negative) were randomly chosen.
Ethics oversight	Collection of tumors (fresh frozen) from BC patients was reviewed and approved by the ethics committee of the Regina Elena National Cancer Institute (IFO1270/19) and written informed consent was obtained from all patients.<br>Concerning serum samples collection in the context of the randomized study reported by Campagnoli et al. 2012 (PMID: 22607767), the study was approved by the institutional review board and ethical committee of all collaborating institutes, as stated in the manuscript.

Note that full information on the approval of the study protocol must also be provided in the manuscript.

Clinical data

Policy information about clinical studies

All manuscripts should comply with the ICMJE guidelines for publication of clinical research and a completed CONSORT checklist must be included with all submissions.

Clinical trial registration	N/A
Study protocol	N/A

Data collection	N/A
Outcomes	N/A
